# Anti-Niemann Pick C1 Single-Stranded Oligonucleotides with Locked Nucleic Acids Potently Reduce Ebola Virus Infection *In Vitro*

**DOI:** 10.1016/j.omtn.2019.04.018

**Published:** 2019-04-25

**Authors:** Anne Sadewasser, Erik Dietzel, Sven Michel, Michael Klüver, Markus Helfer, Tamara Thelemann, Richard Klar, Markus Eickmann, Stephan Becker, Frank Jaschinski

**Affiliations:** 1 Secarna Pharmaceuticals, GmbH & Co. KG, 82152 Planegg/Martinsried, Germany; 2 Deutsches Zentrum für Infektionsforschung (DZIF), Institut für Virologie, Philipps-Universität, 35043 Marburg, Germany

**Keywords:** Ebola virus, antisense oligonucleotide, NPC1, LNA

## Abstract

Ebola virus is the causative agent of Ebola virus disease, a severe, often fatal illness in humans. So far, there are no US Food and Drug Administration (FDA)-approved therapeutics directed against Ebola virus. Here, we selected the host factor Niemann-Pick C1 (*NPC1*), which has been shown to be essential for Ebola virus entry into host cytoplasm, as a therapeutic target for suppression by locked nucleic acid-modified antisense oligonucleotides. Screening of antisense oligonucleotides in human and murine cell lines led to identification of candidates with up to 94% knockdown efficiency and 50% inhibitory concentration (IC_50_**)** values in the submicromolar range. Selected candidate oligonucleotides led to efficient NPC1 protein knockdown *in vitro* without alteration of cell viability. Furthermore, they did not have immune stimulatory activity in cell-based assays. Treatment of Ebola-virus-infected HeLa cells with the most promising candidates resulted in significant (>99%) virus titer reduction, indicating that antisense oligonucleotides against *NPC1* are a promising therapeutic approach for treatment of Ebola virus infection.

## Introduction

Ebola virus (EBOV), as well as four other filoviruses—Bundibugyo virus (BDBV), Sudan virus (SUDV), Marburg virus (MARV), and Ravn virus (RAVV)—are causative agents of severe disease in humans, such as severe hemorrhagic fever, and are often associated with high morbidity and mortality rates.^1^, ^2^, ^3^ These viruses belong to the family *Filoviridae* of non-segmented negative-strand RNA viruses and are biosafety level 4 pathogens transmitted by contact with body fluids, fomites, and droplets from infected patients. Filoviruses are considered a significant threat to public health and global security because of their pandemic potential and the risk of being used as a bioweapon.^1^, ^4^, ^5^, ^6^ Therefore, accelerated efforts in the development of therapeutics is a key objective in the filovirus research community, especially since the 2013–2016 EBOV disease (EVD) epidemic in Western Africa. No vaccines or therapeutic agents with final US Food and Drug Administration (FDA) approval are currently available, and supportive care remains the standard for Ebola virus disease treatment. However, to reduce EBOV spread and the pandemic risk of the current outbreak in Democratic Republic of the Congo (750 confirmed cases and 449 confirmed deaths, as of February 9, 2019) (https://www.who.int/ebola/situation-reports/drc-2018/en/) use of rVSV-ZEBOV Ebola vaccine, as well as antiviral drugs and antibodies against EBOV, have been temporarily approved (https://www.who.int/ebola/drc-2018/faq-vaccine/en/, https://www.who.int/ebola/drc-2018/treatments-approved-for-compassionate-use/en/).

Filovirus particles have a uniform diameter of 80 nm and variable lengths. A single transmembrane glycoprotein (GP), consisting of two subunits, GP1 and -2, is inserted into the virus envelope as a trimeric complex. GP mediates cell attachment and endocytosis by binding to attachment proteins of the host cell.^7^, ^8^ In late endosomes, the host cysteine proteases cathepsin-B and -L cleave and remove large C-terminal regions of the GP1 subunit,^8^, ^9^ thereby unmasking a binding site for the host factor Niemann-Pick C1 (NPC1). This cholesterol transport protein has been shown to be an essential host factor^10^, ^11^ and endosomal entry receptor for filoviruses.^12^, ^13^ In cooperation with Niemann-Pick C2 (NPC2), NPC1 is an endosomal transmembrane protein that mediates transport of luminal cholesterol across the endosomal and lysosomal membrane for dispersal to other cellular compartments.^14^, ^15^ Loss-of-function mutations in *NPC1* or *NPC2* cause a rare and often fatal hereditary neurovisceral disorder in humans.^16^, ^17^ Over time, patients with NPC disease accumulate cholesterol and glycosphingolipids in various tissues and organs, leading to neurological dysfunction and organ failure. Herbert et al.^18^ demonstrated that *Npc1*-deficient mice are completely protected from EBOV infection and free of replicating virus. These results strongly implicate the NPC1 protein as a direct mediator of filovirus infection *in vivo*. It has been reported that NPC1 inhibition by U18666A, an amphipathic steroid, as well as the EBOV-specific antiviral compound 3.47 significantly inhibit filovirus replication by interfering with viral entry^10^, ^11^ *in vitro* and *in vivo*.^18^ However, both compounds have not yet reached clinical trials.

Besides small molecules and therapeutic antibodies, oligonucleotide-based gene expression inhibitors have developed into fully accepted therapeutics. The majority of compounds progressing through clinical trials^19^, ^20^, ^21^ are either antisense oligonucleotides (ASOs), comprising single-stranded DNA-like molecules that recruit endogenous RNase H for target mRNA degradation or small-interfering RNAs (siRNAs) that work through the RNA-induced silencing complex (RISC). ASOs typically have a length of 12–21 nucleotides (nt). In our study, nucleotides were joined via phosphorothioate (PTO) linkages. The phosphorothioate linkage substitutes a sulfur atom for a non-bridging oxygen. This modification renders the internucleotide bond resistant to nuclease degradation and enhances plasma-protein binding while retaining the ability to direct RNase H activity in the cell.^22^, ^23^ In addition, ribose moieties in the flanks of the oligonucleotide are modified by an extra bridge connecting the 2′ oxygen and 4′ carbon. This modification locks the conformation of the ribose, conferring high affinity to the RNA. Therefore, this modification is termed the “locked nucleic acid (LNA) modification.”^24^, ^25^

Here, we demonstrate the potency of LNA-containing ASO (LNA-ASO) molecules targeting mRNA of the host factor *NPC1*, which is essential for filovirus replication. We show that *NPC1*-specific ASOs efficiently reduce viral replication in cultured cells without affecting cell viability or inducing immune-stimulatory responses.

## Results

### Selection of ASOs Targeting Host Factor Niemann-Pick C1

**Figure 1:**
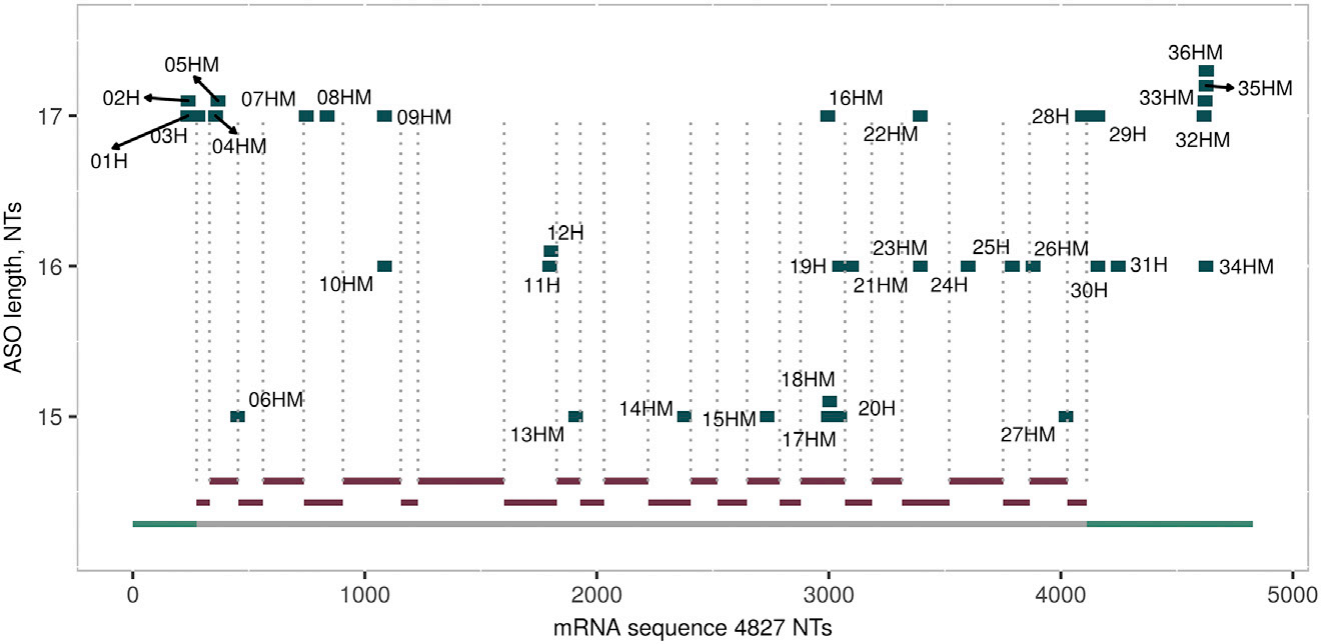
ASO Distribution on Human *NPC1* mRNA All ASOs are depicted according to their location on the human *NPC1* mRNA along the x axis. Distinct exons (red) and UTRs (green) are shown in the bottom part of the figure. The lengths of the ASOs are indicated on the y axis.

*NPC1*-specific 15-, 16- and 17-mer ASOs were selected based on human NCBI reference sequence (accession number GenBank: NM_000271.4) (Table S1). The main criterion for sequence selection was selectivity, to avoid undesired off-target effects. Several sequences were completely cross-reactive to murine *Npc1*, several had one or more mismatches. ASO length, LNA modification pattern, and localization of ASO binding sequence on human *NPC1* mRNA are depicted in Tables 1 and S1 and Figure 1.

**Table 1:** Sequence and Modification of the ASOs 05HM, 28H, and Neg1

Name	mRNA Binding Sequence	Position	Length	Sequence
05HM	GGAGAGTGTGGAATTGC	359	17	+G*+C*+A*A*T*T*C*C*A*C*A*C*T*C*+T*+C*+C
28H	AGCGCGAACGGCTTCTA	4,086	17	+T*+A*+G*A*A*G*C*C*G*T*T*C*G*C*+G*+C*+T
Neg1	N/A	N/A	18	+C*+G*+T*T*T*A*G*G*C*T*A*T*G*T*A*+C*+T*+T

Depicted are name of ASO, mRNA binding sequence, position on mRNA, ASO length as well as ASO sequence and modification: LNA (+) and/or phosphorothioate (*). Human-specific ASOs (H) as well as cross-reactive ASOs targeting both, human and murine *NPC1* (HM), were selected.

### *NPC1* ASOs Efficiently Reduce *NPC1* mRNA Expression in Human and Murine Cell Lines

**Figure 2:**
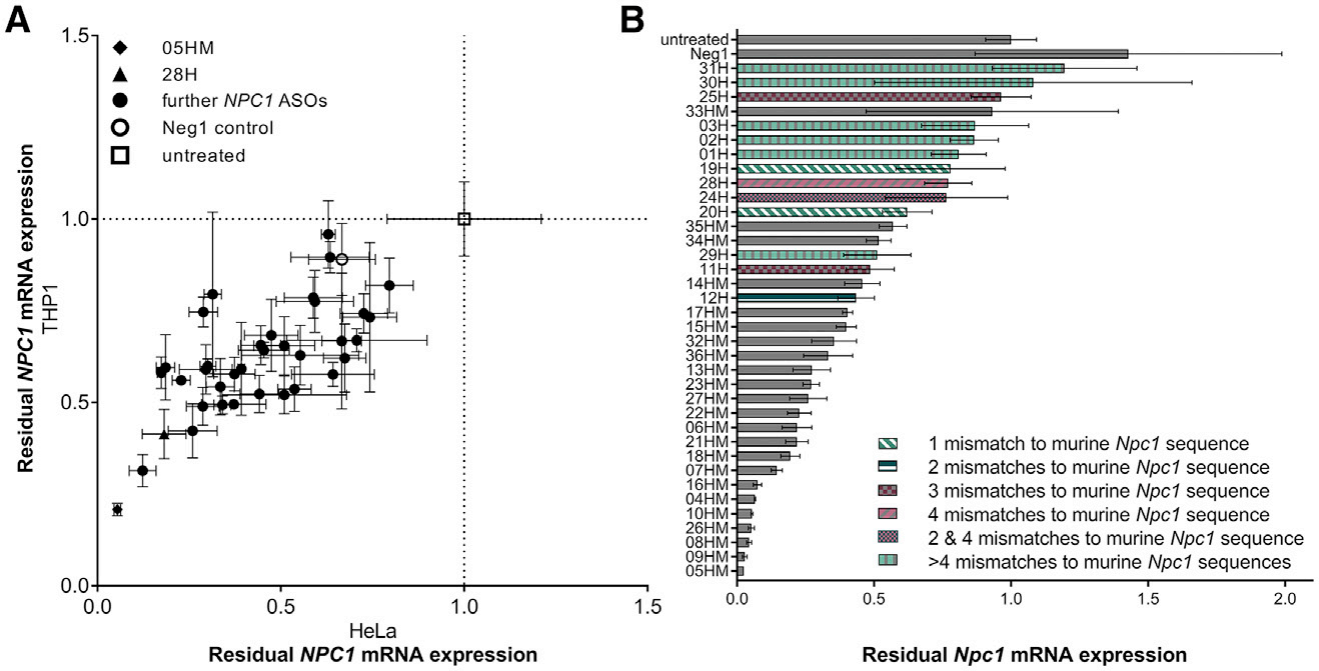
Screening of *NPC1*-Specific LNA-ASOs in Human and Murine Cell Lines (A and B) Human HeLa and THP1 cells (A) as well as murine cell line 4T1 (B) were treated with 10 μM of ASOs. After 3 days cell lysates were used to determine *NPC1* and *HPRT1* mRNA levels. (A) Shown is the correlation of residual *NPC1* mRNA expression (means and SD of triplicate wells) after treatment in HeLa (x axis) versus THP1 cells (y axis). Values were normalized to *HPRT1* and relative to the untreated control (set as 1; empty square). ASO 05HM is indicated as a filled diamond, ASO 28H as a filled triangle, Neg1 as an empty circle, and other *NPC1*-specific ASOs as filled circles. (B) Residual *Npc1* mRNA expression in 4T1 cells after treatment with respective *NPC1*-specific ASOs or negative control Neg1. Values were normalized to *Hprt1* and are shown relative to the untreated control (set as 1). Error bars show SD (triplicate wells). The number of mismatches to murine *Npc1* sequence of human specific ASOs (H) are shown in different colors and patterns.

**Figure 3:**
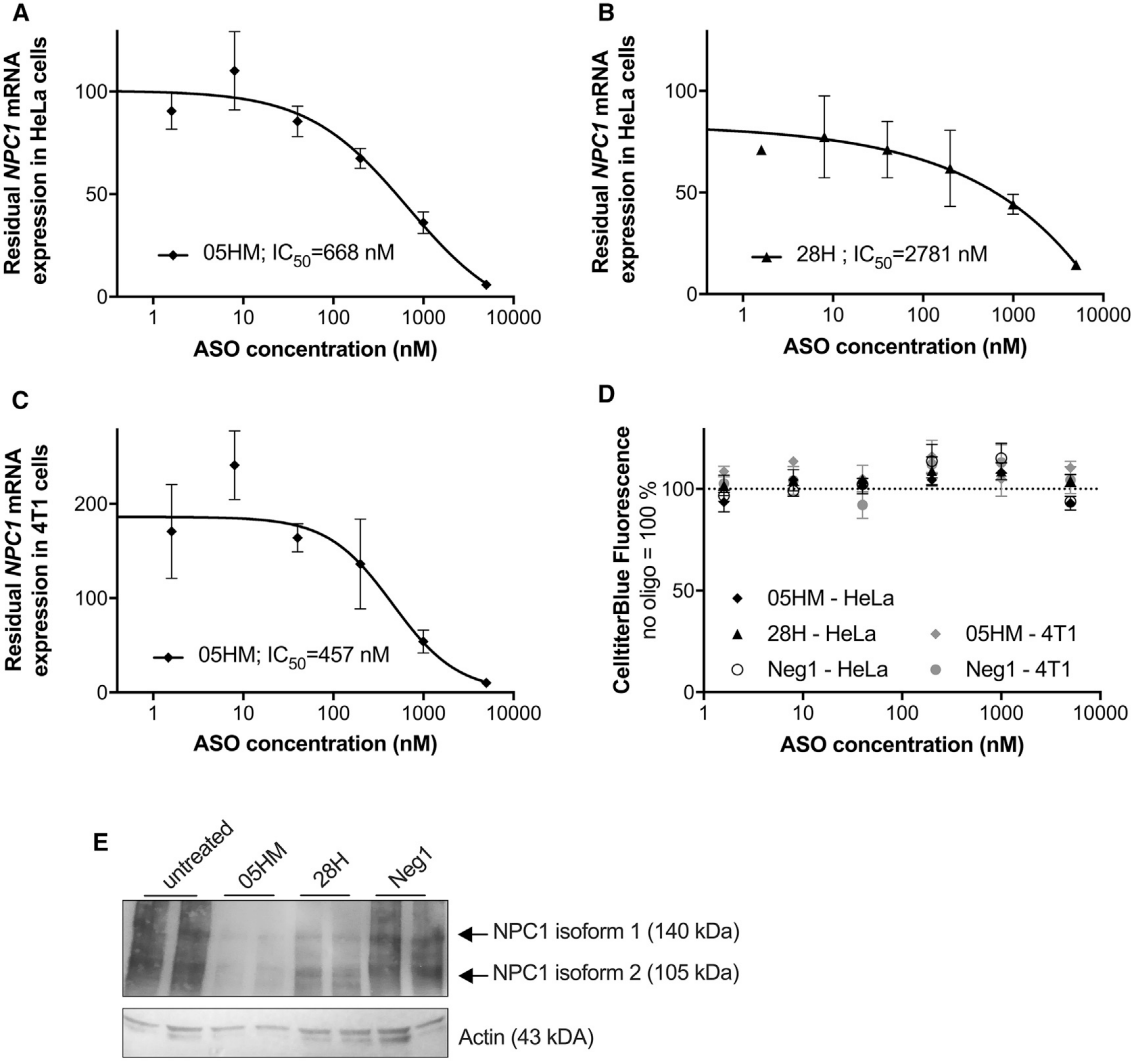
IC_50_ Determination and Protein Knockdown Efficacy of ASOs 05HM and 28H (A–D) HeLa and 4T1 cells were used to generate dose-response curves by treating them with different concentrations of ASO 05HM (A and C) and 28H (B), respectively. On day 3, cell viability was determined using a CellTiter Blue Assay Kit (D). Then, cells were lysed and *NPC1* and *HPRT1* mRNA levels were determined. Values were normalized to the housekeeping gene *HPRT1* and are shown relative to untreated cells (set as 100). IC_50_ values were calculated using Prism 6 (GraphPad Software). Data are means and SD (triplicate wells). (E) HeLa cells were treated twice for 3 days with 10 μM ASO 05HM, 28H, negative control oligonucleotide Neg1 or were left untreated. On day 6, cells were lysed, and lysates were analyzed for NPC1 and Actin protein expression using SDS-PAGE and immunoblot analysis in duplicate wells.

The *in vitro* activity of the 36 *NPC1*-specific ASOs was evaluated in two human and one murine cell line endogenously expressing *NPC1* mRNA. After treating these cells with LNA-ASO without using a transfection reagent,^26^ the level of *NPC1* mRNA was measured after 3 days of treatment. Human HeLa and THP-1 cells were used as cell lines for screening, as both cell lines are susceptible to EBOV infection. *NPC1*-specific ASOs led to reduced *NPC1* mRNA expression levels in both human cell lines with correlating efficacies (Figure 2A). As expected, cross-reactive ASOs having full complementarity to both human and murine *NPC1* mRNA were more efficient in murine 4T1 cells than ASOs that are human-specific and have mismatches to the murine target (Figure 2B). Therefore, an increased number of mismatches of the human-specific ASOs to the murine *Npc1* sequence resulted in decreased efficacy in murine 4T1 cells (Figure 2B). In all three cell lines, the human-mouse cross-reactive ASO 05HM was the most efficient candidate with 95% (HeLa), 79% (THP-1), and 98% (4T1) *NPC1* mRNA knockdown, while the human-specific ASOs 28H and 29H were among the most potent ASOs in human cells, but had poor activity in murine cells (Figure 2). To test dose-dependence of effects, HeLa and 4T1 cells were exposed to increasing concentrations of ASO 05HM and 28H. Endogenous mRNA levels were evaluated after 3 days of treatment with ASOs, and the 50% inhibitory concentration (IC_50_) for the inhibition of *NPC1* expression was determined (Figures 3A–3C). As already indicated by the aforementioned screening results, ASO 05HM (IC_50_ = 668 nM) was more potent in the HeLa cells than was ASO 28H (IC_50_ = 2,781 nM; Figures 3A and 3B). In the murine cell line 4T1, the cross-reactive ASO 05HM was even more effective (IC_50_ = 457 nM; Figure 3C). Notably, treatment with ASOs did not affect cell viability at any concentration (Figure 3D). Using immunoblot analysis, knockdown efficacy on protein level was evaluated and confirmed in HeLa cells, treated twice for 3 days with ASO 05HM and 28H (Figure 3E). Both ASOs clearly reduced NPC1 protein levels compared with untreated cells or with cells treated with control Neg1 that is not complementary to any human or murine RNA (Figure 3E).^27^ Again, treatment of HeLa cells with ASO 05HM resulted in a more pronounced NPC1 knockdown than incubation with ASO 28H (Figure 3E).

Taken together, these data demonstrate the potential of the *NPC1*-specific LNA-ASOs for efficient knockdown of *NPC1* gene expression in human and murine cell lines.

### Selected *NPC1*-Specific LNA-ASOs Do Not Cause Adverse Effects *In Vitro*

Immune activation leading to cytokine release is characteristic of therapeutic oligonucleotides, either as an unwanted side effect or intended pharmacology. This immune activation is mediated by pattern recognition receptors, such as the Toll-like receptors (TLRs). Binding of immune stimulatory ligands, e.g., bacterial DNA or immune stimulatory oligonucleotides, with or without nonmethylated CpG dinucleotides,^28^ results in TLR activation. As immune activation can lead to a severe, possibly life-threatening, condition of excessive cytokine release,^29^ the selected LNA-ASOs 05HM and 28H were analyzed for their potential to activate TLR9 and to induce cytokine release in human cells, enabling a safety assessment for future clinical studies.

**Figure 4:**
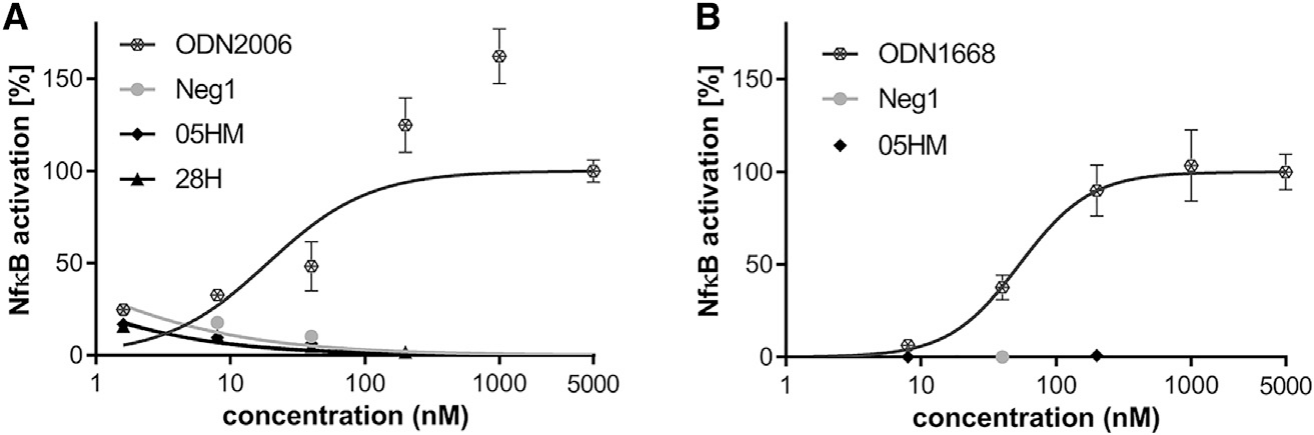
*NPC1*-Specific ASOs 05HM and 28H Did Not Activate TLR9 Signaling (A) HEK-Blue hTLR9 cells were treated with ODN2006 or LNA-ASOs 05HM and 28H, respectively, with the indicated concentrations. After 20 h SEAP reporter activity was measured at 620 nm, using a microplate reader. (B) HEK cells expressing a mouse Tlr9 Nf-кb luciferase reporter plasmid were treated with the indicated concentrations of ODN1668 or LNA-ASO 05HM. After 20 h, the cells were treated with ONE-Glo EX reagent, and luminescence was measured at 560 nm. Values were normalized to untreated cells and are means with SD (triplicate wells).

To assess the potential of *NPC1*-specific ASOs to activate TLR9-mediated signaling, an HEK-Blue hTLR9 SEAP reporter assay was used to measure activation of nuclear factor-kappa light-chain enhancer of activated B cells (NF-кB) induced by TLR9. In contrast to the human TLR9 agonist CpG ODN2006, neither ASO 05HM nor ASO 28H triggered human NF-кB activation (Figure 4A). Murine Nf-кb was also not induced by treatment with cross-reactive ASO 05HM tested in stably transfected HEK-mTlr9_Nf-кb-LUC cells (Figure 4B), whereas the murine Tlr9 agonist ODN1668 induced a considerable dose-dependent response.

**Figure 5:**
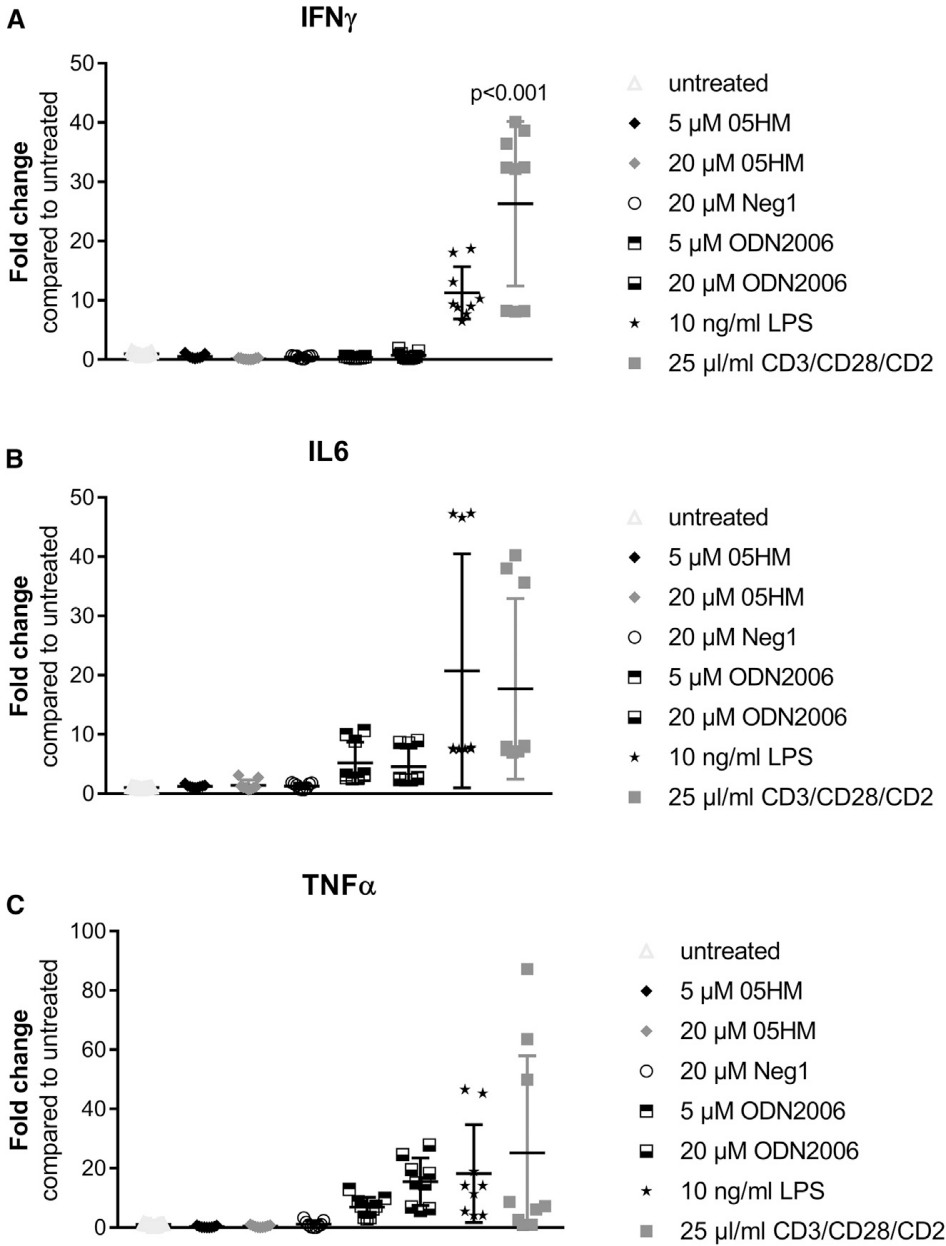
*NPC1*-Specific ASO 05HM Did Not Stimulate Cytokine Release from Treated PBMCs (A–C) PBMCs from three different donors were treated with oligonucleotides and immune stimulatory agents (each condition in triplicate wells), with the indicated concentrations. The third day after treatment, supernatants were harvested and used for determination of cytokine release using ELISA: (A) IFNγ, (B) IL6, and (C) TNFα. Values were displayed as fold changes compared to untreated. Means and SDs were also indicated. ANOVA test was used to test for significant differences and p values were determined using Dunnett’s test in GraphPad Prism 7.04 Software.

TLRs are expressed by numerous cells of the immune system, such as B lymphocytes, monocytes, natural killer (NK) cells, keratinocytes, melanocytes, and plasmacytoid dendritic cells (pDCs). The peripheral blood mononuclear cell (PBMC)-based assay is well established for determination of immune activation by different drugs, including TLR ligands and oligonucleotides.^28^, ^30^, ^31^ Here, PBMC isolated from leukocytes preparations of three different donors were used. In contrast to pattern recognition receptor agonists, ODN2006, lipopolysaccharide (LPS), or immune stimulatory CD3/CD28/CD2, the ASO 05HM failed to trigger a cytokine response (Figure 5).

These data support that the most potent ASOs, 05HM and 28H, do not trigger a host innate immune response.

### *NPC1*-Specific LNA-ASOs Efficiently Inhibit EBOV Replication *In Vitro*

**Figure 6:**
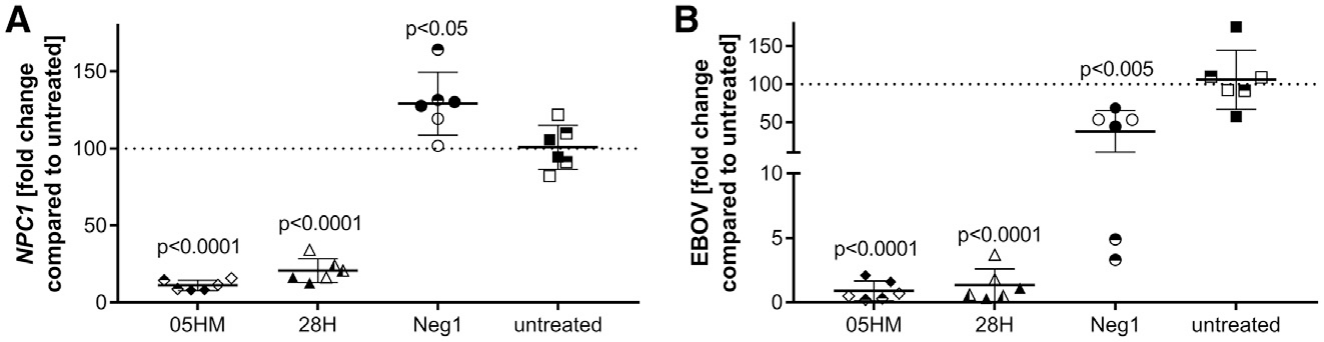
*NPC1*-Specific ASOs 05HM and 28H Specifically Inhibit EBOV Replication (A and B) HeLa cells pretreated with the respective ASO were infected with EBOV at an MOI of 0.01. At 1 day after infection (p.i.), *NPC1* (A) and EBOV (B) levels were quantified by RT-qPCR and normalized to the internal control α-tubulin. Shown is the fold change compared to untreated control (set at 100), which was calculated using the 2^−ΔΔCt^ method. Error bars show SD (n = 3, each in duplicate). Duplicates are labeled with identical symbol shapes. ANOVA was used to test for significant differences and p values were determined using Dunnett’s test, in GraphPad Prism 7.04 Software. See also Figure S2.

LNA-ASOs 05HM and 28H were then tested for antiviral activity during EBOV infection. To ensure efficient downregulation of endosomal NPC1 protein (Figure 3E), HeLa cells were treated twice with ASO 05HM and 28H for 3 days. Then, cells were infected with EBOV at an MOI of 0.01 and incubated for 24 h in medium containing the respective ASOs. Compared to untreated cells, *NPC1* mRNA was significantly reduced (by about 89% [05HM] and 80% [28H]; Figure 6A), whereas EBOV virus genome copies were decreased by about 99% (05HM) and 98% (28H), 1 day after infection, as quantified by qPCR (Figure 6B). Treatment with the control oligonucleotides Neg1 (Figure 6A; Figure S2A), Neg1B, or S5 (Figure S2A) did not reduce *NPC1* mRNA levels. However, EBOV replication was also decreased (by about 62% [Neg1, Figure 6B] and 70% [Neg1B and S5; Figure S2B]), most likely due to the backbone-mediated effects of phosphorothioate-modified oligonucleotides (Figure 6B; Figure S2A).

Treatment with phosphorothioate-modified non-targeting oligonucleotides reduced EBOV replication by 62-70% *in vitro*. Furthermore, oligonucleotides specifically targeting NPC1, an essential receptor of EBOV cell entry, led to a more potent inhibition of EBOV replication—up to 99%.

## Discussion

EBOV causes a severe, often fatal illness in humans, is transmitted to people from wild animals, and spreads in the human population through human-to-human transmission.^32^ Case fatality rates of Ebola virus disease have varied from 25% to 90% in past outbreaks, with an average of around 50%.^33^, ^34^ There are two distinct paths for potential EBOV treatments: post-exposure prophylaxis and treatment of symptomatic patients. Both have different challenges, but a common strategy may be to limit virus replication, to allow the adaptive and innate immune systems time to fight the infection.^35^, ^36^ A limitation for ASO-based antiviral strategies directly targeting the virus may be the RNase H1-dependent mode of action of gapmer ASOs. Filoviruses replicate in the host cytoplasm and do not require the nucleus.^37^ However, the cellular compartment of ASO-mediated RNA degradation is a controversial issue within the ASO community.^38^, ^39^, ^40^, ^41^ We and others showed that ASOs targeting EBOV mRNA could efficiently block viral transcription or replication in cell-free^42^ and reporter gene assays (Figures S1A and S1B). In reporter-based assays, transcription of the reporter genes takes place in the nucleus. However, EBOV-specific ASOs were not capable of inhibiting viral propagation in Huh7 cells, with or without the use of a transfection reagent (Figures S1C and S1D). These results indicate, that the predominant location of RNase H1-mediated target RNA degradation is the nucleus that is, as previously mentioned, bypassed during EBOV replication. The use of ASO approaches aimed at sterically blocking viral translation in the cytoplasm could avoid this issue. However, translation-blocking approaches are much less efficient compared to RNase-H1-dependent mechanisms. Whereas one translation-blocking ASO-molecule is needed to repress translation of one target mRNA molecule, one RNase H1-depending ASO could mediate degradation of many individual RNA molecules. Therefore, much higher doses would be necessary for a translation-blocking approach in the cytoplasm. The antiviral efficacy of translation-blocking approaches was suggested by Chery et al.^42^ indirectly by use of reporter-based assays. However, direct evidence for antiviral effects such as copy number determination of an EBOV isolate after ASO treatment is missing. A fundamental disadvantage of approaches targeting EBOV directly is the error-prone viral polymerase of RNA viruses that enables incorporation of the mutations that facilitate resistance against antiviral drugs. Furthermore, antiviral approaches have not been capable of overcoming strain-specific differences, so far. Although the efficacy of antibody cocktails, such as ZMapp, for EBOV in nonhuman primates is clear, a future challenge will be to identify similar treatments for other filoviruses.^43^

For the aforementioned reasons, targeting an essential human host factor for viral replication may be one of the most promising approaches for fighting EBOV. An essential host factor for filovirus replication *in vitro* and *in vivo* is the cholesterol transport protein NPC1. This endosomal entry receptor has been shown to mediate cytoplasmic release of viral ribonucleoproteins,^10^, ^13^ making it an ideal target for blocking filovirus replication.

Various small molecule therapeutics against host factor NPC1 have been described, some with demonstrated efficacy against filovirus replication in mice.^43^ However, those molecules targeting NPC1 did not reach clinical trials, so far, probably because of the described toxic effects^44^ or the need for further mechanistic characterization, as well as improvement of compounds.^43^ The limited success of the available NPC1 inhibitors in protecting mice from EBOV challenge highlights the need for new molecules or approaches to target NPC1 *in vivo.*^18^

A unique advantage of ASOs targeting essential host factors for filovirus replication, particularly in the context of emergency prophylactic or post-exposure therapeutics, is their target specificity, cost-effectiveness, and fast generation. Mode of action, uptake, biodistribution, pharmacokinetics, and safety issues have been extensively studied.^24^, ^26^, ^45^, ^46^, ^47^, ^48^, ^49^, ^50^

In the present study, a total of 36 ASOs was designed for the initial screen broadly covering the *NPC1* mRNA sequence. Fifteen-, 16-, and 17-mer ASOs were tested, and two ASOs, comprising 17 nt, were selected for further experiments. As recommended by the Oligonucleotide Safety Working Group,^51^ an extensive *in silico* approach was used to avoid suppression of off-target genes. The bioinformatic analysis revealed no perfect match to any exonic or intronic off-target sequence. Even allowing one mismatch, still no off-target hit was detected in the NCBI RefSeq data base. This off-target characteristic is well in the range of the characteristic of other ASOs recently published in the field.^42^, ^52^ This should also decrease risk of generation of ASO-generated RNA fragments, which, as Dieckmann et al.^45^ speculated, could cause toxicity.

ASOs have been repeatedly described to stimulate immune activation.^22^, ^53^, ^54^, ^55^, ^56^ Reasons for immune stimulatory activities were reported to be nonmethylated CpG dinucleotides within the oligonucleotide sequence, as well as the stabilizing phosphorothioate backbone.^57^, ^58^, ^59^ Studies using Tlr9-deficient mice demonstrated that this Tlr subtype is essential for the effects that are mediated by bacterial DNA or CpG oligonucleotides.^60^ Since expression, ligand preference, and function of pattern-recognition receptors is highly species specific,^61^ cytokine release in humans is hard to predict in animal studies. The human TLR9 is expressed in B cells and pDCs.^61^, ^62^, ^63^ Both cell types are stimulated by CpG oligonucleotides to upregulate cell surface costimulatory molecules and to secrete a variety of cytokines.^64^, ^65^ These effects can lead to indirect activation of other cell populations, such as monocytes and NK or T cells.^65^ The *in vitro* TLR9 assays as well as the PBMC *ex vivo* test used in this study are therefore helpful in deselecting ASOs with immune stimulatory potential early in the screening and compound characterization process, to prevent unexpected harmful effects in clinical development. In contrast to the CpG oligonucleotides ODN2006 and ODN1668, which were used as positive controls, both of the selected *NPC1*-targeting ASOs 05HM and 28H activated neither human nor murine TLR9 (Figure 4), nor did ASO 05HM stimulate cytokine release in treated PBMCs (Figure 5). These results are in line with the findings of Vollmer and colleagues,^28^ who demonstrated that LNA modification of ASOs significantly decreases the immune stimulatory effects of ASOs. Our bioinformatics analysis enabled the selection of cross-reactive ASOs that target human as well as murine *NPC1* mRNA for screening in human and murine cell lines. Again, the benefit of our rational ASO design was confirmed, as cross-reactive ASOs were effective in both species *in vitro* (Figure 2). As expected, a decreased knockdown of murine *Npc1* by human-specific ASOs with no 100% complementarity to the murine mRNA was observed in murine cells. Thereby, a higher number of mismatches to the murine *Npc1* sequence was associated with decreased activity in murine cells (Figure 2B). In *Npc1*^*−/−*^ mice, it was previously demonstrated that the Npc1 protein acts as a direct mediator for EBOV infection *in vivo*.^18^ Since ASO 05HM targets human and murine *NPC1*, it is also a suitable tool for testing *in vivo* efficacy against different filovirus strains in mice.

*NPC1* ASO screening in two different human cell lines resulted in strong correlation of ASO activity, which enabled reliable selection of candidate ASOs. Consistently, ASO 05HM was the most effective candidate during single-dose screens in one murine and two human and cell lines (Figure 2), during IC_50_ determination (Figures 3A–3C), protein knockdown (Figure 3E), and the infection assay in HeLa cells (Figure 6). All experiments were performed by using a method called “gymnosis” without using a transfection reagent.^26^ The pattern of gene silencing of *in vitro* gymnotically delivered oligonucleotides correlates particularly well with *in vivo* silencing and is therefore of particular significance for drug discovery.^26^ Recently, Chery and colleagues^42^ also reported on efficient knockdown of *NPC1* by use of target-specific ASOs. However, these ASOs were delivered into cells using lipofection and may have divergent characteristics *in vivo*.

Macrophages, monocytes, and dendritic cells are the primary target cells during acute EBOV infection, and several organs, such as liver, kidneys, and spleen, show high viral loads during the course of infection,^66^, ^67^, ^68^, ^69^ These cell types and organs are also good targets for phosphorothioate-modified ASOs which have a preferred biodistribution to kidney, liver, and immune cells and are not capable of crossing the blood-brain barrier.^46^ Therefore they cannot affect the transport of cholesterol in neurons within the CNS, which could cause a Niemann-Pick disease-like phenotype.

Treatment with *NPC1*-specific ASO resulted in significantly decreased EBOV replication (>99%; Figure 6B). Similar results were obtained by Chery and colleagues^42^ by use of a VSVluc-EboV GP reporter virus. Reduced luciferase expression up to 80% was detected after transfection of HeLa cells with an *NPC1*-specific ASO. However, neither was the immune stimulatory potential of the selected ASO tested nor was a negative control ASO included to test for potential unspecific effects. Therefore, whether the observed decrease in reporter virus replication is a backbone-mediated or a specific effect caused by *NPC1* knockdown cannot be distinguised.^42^ In the present study, the control oligonucleotides clearly diminished the amount of EBOV genome copies 24 h after infection, though to a lesser extent (Figure 6B; Figure S2B). In addition to their sequence-specific functionality, single-stranded oligonucleotides are polymers with polyanionic characteristics that are largely conserved, regardless of their nucleotide sequence. Phosphorothioation of oligonucleotides confers increased hydrophobicity and has been shown to specifically mediate antiviral activity in a manner independent of the increased nuclease stability present with this modification.^70^, ^71^ Phosphorothioates with broad-spectrum activity against viral and other infectious diseases are also called “nucleic acid polymers” (NAPs).^72^ The structure-function relationship of the antiviral activity of NAPs, as well as their molecular mechanism of action, was first elucidated in a study describing the specific antiviral effects of NAPs during the entry of HIV-1.^71^ In this study, the entry inhibition effect of NAPs was shown to be independent of sequence, but dependent on size.^71^ A large amphipathic protein domain in the viral gp41 glycoprotein is required for interaction with NAPs and conserved in class I fusion GPs from many other viruses susceptible to antiviral polymers. Following on the findings in the initial study in HIV-1, NAPs were subsequently shown to have the same sequence-independent and phosphorothioate as well as length-dependent antiviral effects in other viruses with class I fusion GPs, including herpesviruses, cytomegalovirus, influenza virus, and lymphocytic choriomeningitis virus.^73^, ^74^, ^75^, ^76^, ^77^ Meanwhile, the company Replicor develops antiviral drug candidates against hepatitis B and D viruses based on the mechanism by which NAPs inhibit viral propagation.

Interestingly, EBOV GP, which is required for virus entry into the host cell, is also a class I fusion protein.^78^ Keeping this fact in mind, it may be reasonable that, to some extent, phosphorothioated oligonucleotides inhibit EBOV replication, independent of sequence.

These findings could explain the 62-70% reduction of viral replication detected with control phosphorothioates that did not reduce *NPC1* mRNA levels (Figures 6A and 6B; Figures S2A and S2B). However, the specific oligonucleotides 05HM and 28H significantly reduced the EBOV titer after 24 h of infection by 97%–99% compared to non-treated cells (Figure 6B; Figure S2B). This result strengthens our hypothesis that RNase H1-mediated targeting of an essential host factor for virus entry has an additional effect of solely backbone-mediated reduction of EBOV entry, thereby vastly improving therapeutic antiviral potential.

Taken together, the findings in this study demonstrate that knockdown of intracellular receptor *NPC1* by target-specific ASOs is a promising approach for treatment of EBOV infection. Our selected human-mouse cross-reactive *NPC1*-specific ASO may be used in future studies to further investigate the efficacy of *Npc1*-specific ASO against EBOV in mouse models.

## Materials and Methods

### Selection of *NPC1*-Specific ASOs

The human mRNA sequence of *NPC1*, as defined by NCBI accession number GenBank: NM_000271.4, was taken as a basis to design the ASOs. For this sequence (4,827 bp) the possible 15-, 16-, and 17-mer sequence fragments were extracted. All 14,442 fragments were tested for specificity against the complete NCBI RefSeq (https://www.ncbi.nlm.nih.gov/refseq/) and ENSEMBL (http://www.ensembl.org/) databases, by using PatMaN.^79^ Possible matches, including up to three mismatches against any mRNA (RefSeq) or any intron (ENSEMBL), were collected to enable a filtering of those fragments showing the highest specificity. As a cross-reactivity to mice was additionally desired, the sequences were tested against all mouse sequences of both databases, as well.

The main criterion for the selection process was the absence of a perfect match to any human or mice off-target mRNA or intron sequence. In addition, only a limited number of off-target matches allowing up to three mismatches were tolerated. As recommended by the distributor (https://www.qiagen.com/de/shop/pcr/primer-sets/custom-lna-oligonucleotides/?akamai-feo=off&clear=true#productdetails), sequences containing a triple C or triple G pattern were avoided. The melting temperatures of the reverse complements of the remaining sequences were determined *in silico*, considering the effects of LNA modifications. The typical LNA-gapmer format of three modifications at each side^80^, ^81^ was optimized, if there were strong deviations from the internally defined range of an appropriate melting temperature. In the first screen, 23 sequences showing a perfect match to human and mice *NPC1* mRNA and 13 sequences specific only to the human mRNA were used to design ASOs containing phosphorothioate bonds and the optimal LNA pattern.

For screening, IC_50_ determination and analysis of protein knockdown, 40 nmol of ASOs were synthesized by Eurogentec (Cologne, Germany). After selection of two of the most effective candidates, 5 mg of the ASOs 05HM and 28H were purchased from Exiqon (Copenhagen, Denmark) for *in vitro* infection assays. Control oligonucleotides Neg1 and S5 were synthesized by Exiqon (Copenhagen, Denmark), and negative control oligonucleotide Neg1b was purchased from Axolabs (Kulmbach, Germany). ASOs were purified by reverse-phase high-performance liquid chromatography (HPLC) and subsequently lyophilized. After receiving the ASOs, they were solubilized in diethyl-pyrocarbonate (DEPC)-treated H_2_O to a concentration of 1 mM.

### Screening of *NPC1*-Specific ASOs

HeLa (ACC 57; DSMZ, Braunschweig, Germany;), THP-1 (ACC 16; DSMZ), and 4T1 (CRL-2539; ATCC-LGC Standards, Wesel, Germany) cells were cultured in DMEM GlutaMAX (32430027; Gibco, Life Technologies, Darmstadt, Germany) supplemented with 10% fetal calf serum (FCS; 10270106; Gibco), 1 mM sodium pyruvate (11360070; Gibco), and 1× antibiotic-antimycotic (15240062; Gibco).

The cells were detached using trypsin (25200056; Gibco), seeded into 96-well plates at 6,000 (HeLa), 20,000 (THP-1), and 5,000 (4T1) cells per well and treated with the *NPC1* ASO compounds on day 0. Treatments were done at a final concentration of 10 μM with triplicate experiments for each compound. A control oligonucleotide, Neg1, was used at a final concentration of 10 μM with triplicate experiments. On day 3, cells were lysed, and mRNA levels were measured according to the manufacturers’ instructions, using QuantiGene Singleplex Gene Expression Assay (QS0011; Life Technologies, Darmstadt, Germany) and the following probe sets: human probe sets specific for *NPC1* (SA-10502; Life Technologies) and the housekeeping gene *HPRT1* (SA-10030; Life Technologies) or probe sets specific for murine *Npc1* (SB-12805; Life Technologies) and *Hprt1* (SB-15463; Life Technologies).

Residual *NPC1* mRNA expression levels were calculated by comparing the *NPC1* values normalized to *HPRT1* in the ASO-treated samples with that measured in the untreated control.

### IC_50_ Determination

HeLa and 4T1 cells were used to generate dose-response curves by treating them with different concentrations (5,000 nM, 1,000 nM, 200 nM, 40 nM, 8 nM, and 1.6 nM) of ASO 05HM (4T1; HeLa) and 28H (HeLa). On day 3 after treatment, cell viability was determined using the CellTiter-Blue Assay (G8081; Promega, Mannheim, Germany) according to the manufacturer’s instructions. Afterwards, the cells were lysed, and mRNA levels were measured according to the manufacturers’ instructions, using the QuantiGene Singleplex Gene Expression Assay (QS0011; Life Technologies) and the following probe sets: human probe sets specific for *NPC1* (SA-10502; Life Technologies) and the housekeeping gene *HPRT1* (SA-10030; Life Technologies) or probe sets specific for murine *Npc1* (SB-12805; Life Technologies) and *Hprt1* (SB-15463; Life Technologies). Values were normalized to the housekeeping gene *HPRT1* and cell control (untreated cells). IC_50_ values were calculated using Prism 6 (GraphPad Software).

### Immunoblot Analysis

HeLa cells were seeded into 12-well plates at 75,000 cells per well and treated twice with 10 μM ASO 05HM and 28H for 3 days each. On day 6, cells were lysed using 100 μL RIPA buffer (89900; Thermo Scientific, Life Technologies) per well supplemented with Halt Protease Inhibitor Cocktail (1861278; Thermo Scientific). Samples were prepared for SDS-PAGE using 4× Laemmli sample buffer (161-0747; Bio-Rad Laboratories, München, Germany) and proteins were separated using Mini-Protean TGX™ Precast Gels (456-8025; Bio-Rad Laboratories), Precision Plus Protein Western C Standard (161-0376; Bio-Rad Laboratories), and 1× Tris/glycine/SDS (TGS; 161-0732; Bio-Rad Laboratories) according to the manufacturer’s instructions. Polyvinylidene fluoride (PVDF) membrane (162-0177; Bio-Rad Laboratories) was activated in 100% ethanol (5054.4; Carl Roth, Karlsruhe, Germany) and equilibrated in 1× Tris/glycine (TG) buffer (161-0734; Bio-Rad Laboratories). Separated proteins were transferred to the membrane using OmniPAGE mini vertical systems and Semi-dry Blotter (Cleaver Scientific, Warwickshire, United Kingdom) and 10× TG buffer. Protein detection was performed using the Amplified Opti-4CN Substrate Kit (1708238; Bio-Rad Laboratories), according to the manufacturer’s instructions and the following antibody dilutions: NPC1 antibody (1:500, ab55706; Abcam, Cambridge, United Kingdom), Actin antibody (1:500, VMA00048; Bio-Rad AbD Serotec, Puchheim, Germany), goat anti-mouse-horse radish peroxidase (GAM-HRP, 1:1,000, STAR207P; Bio-Rad AbD Serotec).

### TLR9 Reporter Assay

HEK-Blue hTLR9 cells, which were generated by co-transfection of the hTLR9 gene and an optimized SEAP reporter gene into HEK293 cells, were obtained from InvivoGen (hkb-htlr9; Toulouse, France). The SEAP reporter gene was placed under the control of the interferon-beta (IFNβ) minimal promoter fused to five NF-кB and activator protein 1 (AP-1) binding sites. Stimulation with a TLR9 ligand activates NF-кB and AP-1, which induce the production of SEAP. Cells were cultivated in DMEM GlutaMAX (32430027; Gibco, Life Technologies) supplemented with 10% FCS (10270106; Gibco), 1 mM sodium pyruvate (11360070; Gibco), 1× antibiotic-antimycotic (15240062; Gibco), 10 μg/mL Blasticidin (ant-bl-05; InvivoGen) and 100 μg/mL Zeocin (ant-zn-1; InvivoGen). Cells were seeded at 25,000 cells per well in 96-well plates and incubated for 20 h at 37°C and 5% CO_2_. Then, they were treated with ODN2006 (tlrl-2006; InvivoGen) or LNA-ASOs 05HM and 28H, with 5-fold serial dilutions, starting at a concentration of 5,000–1.6 nM. As a control, cells were treated with cell culture medium without addition of oligonucleotides. Each condition was performed in triplicate. Twenty hours after cell treatment, 100 mL Quanti-Blue (QB) Solution (rep-qbs; InvivoGen) was prepared by adding 1 mL of QB reagent and 1 mL of QB buffer to 98 mL of sterile water in a sterile glass bottle or flask. QB Solution was gently mixed and incubated for 10 min at room temperature before it was added to the sample. HEK-Blue-hTLR9 cell supernatants (20 μL per well) were harvested into a fresh 96-well plate and 180 μL QB solution was added to each well. Samples were incubated for 2 h at 37°C, and SEAP activity was determined by measurement of the optical density at 620 nm with a microplate reader.

Stably transfected HEK cells expressing a mouse Tlr9 Nf-кb luciferase reporter plasmid, kindly provided by Prof. Holger Garn, University of Marburg, were used for the murine Tlr9 reporter gene assay. Stimulation with mouse Tlr9 ligands activate Nf-кb which induces the expression of firefly luciferase (*Photinus pyralis*). HEK-mTlr9_ Nf-кb-LUC cells (25,000/well) were plated in a white-walled, 96-well tissue-culture plate in DMEM GlutaMAX (32430027; Gibco, supplemented as described above). Twenty hours after cell seeding, the cells were treated with 5-fold serial dilutions of ODN1668 (tlrl-1668; InvivoGen) or LNA-ASO 05HM, starting at a concentration of 5,000–1.6 nM and incubated for 20 h at 37°C in the incubator. As a control, cells were treated with cell culture medium without addition of oligonucleotides. Each condition was performed in triplicate. After the incubation period, the plates were centrifuged for 5 min at 500 *g*, and the cell supernatants were removed. ONE-Glo EX reagent (50 μL, E8110; Promega) was added to each well, and cells were lysed according to the manufacturer’s instructions. Luminescence was immediately measured at 560 nm.

### Cytokine Release

PBMCs were isolated from leukapheresis products donated by three healthy individuals (Klinikum rechts der Isar, TU München, ethics commission reference: 329/16 S), by density gradient centrifugation. The leukapheresis product was diluted 1:10 with PBS (10010-023; Gibco) and carefully loaded onto 15 mL Biocoll separating solution (L6115; Biochrom, Berlin, Germany) in 50 mL Falcon tubes. Density gradient centrifugation was performed at 800 *g* for 20 min at room temperature with the brake turned off. Afterward, the mononuclear cell layer was collected carefully and transferred into a new 50 mL Falcon tube, and PBS was added to a final volume of 50 mL. After centrifugation at 500 *g* for 5 min at room temperature (brake turned on) supernatants were discarded, and cell pellets were pooled in PBS in a total volume of 50 mL. Cells were counted, divided into aliquots, and stored on liquid nitrogen for further use. PBMCs (400,000) were seeded in RPMI-1640 medium (72400-047; Gibco) supplemented with 10% FCS, 1 mM sodium pyruvate, 1× antibiotic-antimycotic, and 50 U/mL Benzonase (70746-3; Merck, Darmstadt, Germany) per well of a 96-well plate. Afterwards, the cells were treated with RPMI containing either oligonucleotides (5 or 20 μM ASO 05HM or 20 μM negative control Neg1) or immune stimulatory agents: 20 μM ODN2006 (tlrl-2006; InvivoGen), 10 ng/mL LPS (L4391; Sigma Aldrich, Taufkirchen, Germany), or 25 μL/mL CD3/CD28/CD2 (10970; StemCell Technologies, Inc., Grenoble, France). Each condition was performed in triplicate. Treated cells were then rested for 72 h at 37°C and 5% CO_2_. The third day after treatment, 96-well plates were subjected to centrifugation at 2,000 rpm for 10 min at 4°C. Cell supernatants were collected in 96-well plates. For the measurement of IFNγ secretion, 50 μL of cell supernatant was diluted with 50 μL ELISA/ELISPOT diluent. IFNγ measurement was performed with IFNγ Human Uncoated ELISA Kit from eBioscience (88-7316-88; Thermo Fisher Scientific) according to the manufacturer’s protocol. For the measurement of IL6 and TNFα secretion, 50 μL of cell supernatant was diluted with 50 μL Assay Diluent A. IL-6 and TNFα measurements were performed with IL-6 and TNFα ELISA Kits, respectively, from BioLegend (Koblenz, Germany [IL6; 430505] and [TNFα; 430201]), according to the manufacturer’s protocols.

### EBOV Infection Assay

All work with infectious EBOV was performed in compliance with national regulations at the BSL4 Laboratory of the Institute of Virology, Philipps-University, Marburg.

HeLa cells were pretreated in duplicate two times for 3 days by adding medium containing 10 μM of ASOs 05HM and 28H and negative control oligonucleotides Neg1, Neg1B, or S5 or medium only (mock). At 1 h prior to infection, ASOs were removed. Cells were then infected with EBOV Mayinga (NCBI accession number GenBank: AF086833.2) at an MOI of 0.01 for 3 h in the absence of ASOs. Subsequently, the cells were washed to remove unbound input virus and incubated in the presence of 10 μM of the respective ASO. At 24 h after infection, cellular RNA was isolated using the RNeasy Kit (74106; QIAGEN, Hilden, Germany). For virus quantification, EBOV L- or GP-specific primer sets and probes were used for qRT-PCR analysis.^82^ Knockdown of *NPC1* was analyzed via qRT-PCR with *NPC1* specific primers (VHPS-6283; Real-Time Primers, Elkins Park, PA) using the QuantiTect SYBR Green RT-PCR Kit (204143; QIAGEN). Ct values were normalized to the internal control α-tubulin (2^−ΔCt^), and the fold change over mock was calculated using the 2^−ΔΔCt^ method.^83^

### Statistical Analysis

GraphPad Prism 7.04 Software was used for statistical calculations. The efficacy of the ASOs was assessed by one-way ANOVA followed by Dunnett’s multiple-comparison test, to contrast the treatment groups (including control ASOs) with mock control. Differences were considered statistically significant when p < 0.05.

## Author Contributions

The study design was developed by A.S., E.D., M.K., M.H., M.E., S.B., and F.J. The bioinformatic design of the ASOs was created by S.M. The experiments were executed by A.S., E.D., M.K., M.H., T.T., and R.K. Data were evaluated by A.S., M.K., M.H., and E.D. The manuscript was written by A.S., E.D., S.M., and F.J.

## Conflicts of Interest

A.S., S.M., M.H., T.T., R.K. and F.J. are/were employees of Secarna Pharmaceuticals & Co. KG (Planegg, Germany). The rest of the authors declare no competing interests.

## Supplemental Information

Document Sl. Figures S1 and S2, Table S1, and Supplemental Methods

Document S2. Article plus Supplemental Information
