# Supplementary material for: Anti-Niemann Pick C1 Single-Stranded Oligonucleotides with Locked Nucleic Acids Potently Reduce Ebola Virus Infection In Vitro
Source: Mol Ther Nucleic Acids. 2019 Apr 25;16:686–97. doi: 10.1016/j.omtn.2019.04.018 (PMC6529764; doi:10.1016/j.omtn.2019.04.018)
Supplement: Document Sl. Figures S1 and S2, Table S1, and Supplemental Methods [file mmc1.pdf]

OMTN, Volume *16*

Supplemental Information

Anti-Niemann Pick C1 Single-Stranded Oligonucleotides with Locked Nucleic Acids Potently Reduce Ebola Virus Infection *In Vitro*

Anne Sadewasser, Erik Dietzel, Sven Michel, Michael Klüver, Markus Helfer, Tamara Thelemann, Richard Klar, Markus Eickmann, Stephan Becker, and Frank Jaschinski

Supplemental Figures
Fig. S1

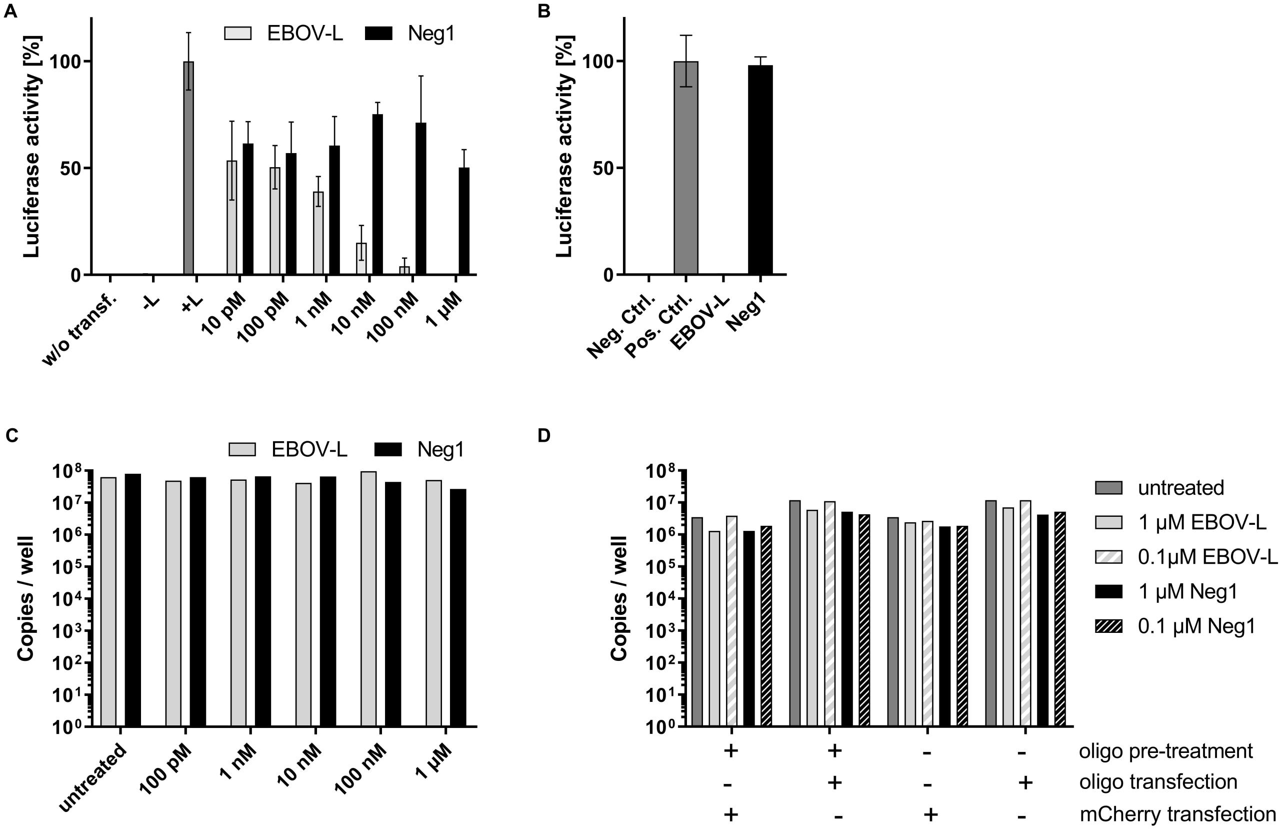

Fig. S2

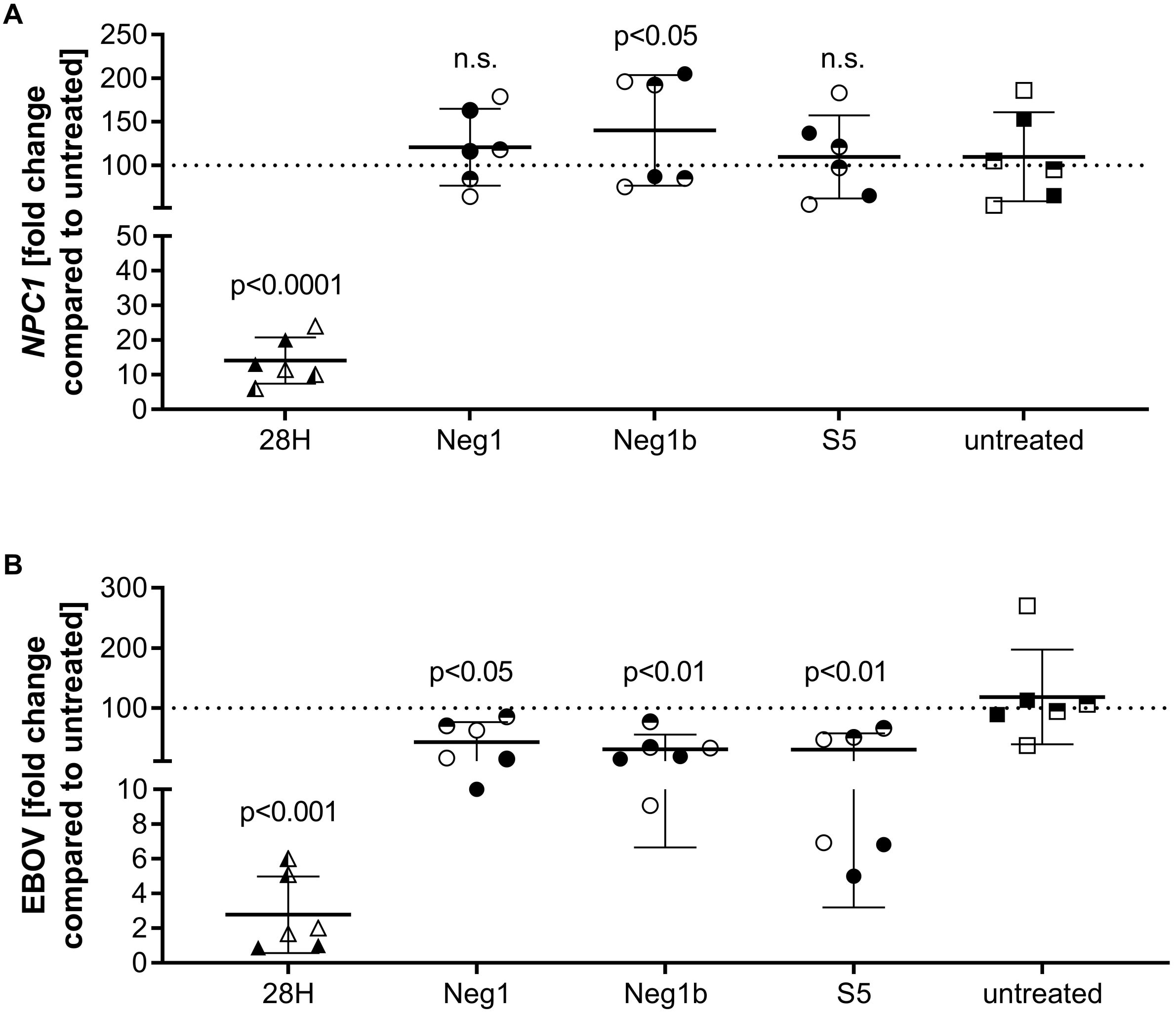
A
NPC1 [fold change compared to untreated]
250
200
150
100
50
40
30
20
10
0
p<0.0001
n.s.
p<0.05
n.s.
28H
Neg1
Neg1b
S5
untreated
B
EBOV [fold change compared to untreated]
300
200
100
10
8
6
4
2
0
p<0.001
p<0.05
p<0.01
p<0.01
28H
Neg1
Neg1b
S5
untreated

Supplemental Figure Legends

Fig. S1. EBOV L-specific ASO shows specific inhibition in transfection-based assays, but not during EBOV infection. (A) After seeding Huh7 cells, triplicate wells were treated with serial dilutions of EBOV L-specific ASO or negative control Neg1. The next day, cells were used for EBOV minigenome assay and transfected with the corresponding plasmids (details see supplemental methods). Three hours post transfection, medium was changed to ASO-containing medium. 24 hours post transfection cells were lysed and used for renilla luciferase assay. The positive control was set to 100 % (n=1). Displayed are means with standard deviations of triplicate wells. (B) After seeding of Huh7 cells, duplicate wells were treated with EBOV L-specific ASO or negative control Neg1. The next day, cells were used for EBOV trVLP Assay (producer cell setting) and transfected with the corresponding plasmids (details see supplemental methods). For normalization of transfection efficiency, a firefly luciferase reporter was added. Four hours post transfection, medium was changed to ASO-containing medium. 24 hours post transfection cells were lysed and used for renilla and firefly luciferase assay. Renilla reporter signals were normalized by use of firefly signals and the positive control was set to 100 % (n=1). Displayed are means with standard deviations of duplicate wells. (C) Huh7 cells were pre-incubated with a serial dilution of EBOV L-specific ASO or negative control Neg1. The next day, cells were infected with EBOV Makona with an MOI of 0.01 for 1 hour. After infection, cells were washed to remove unbound virus and ASO-containing medium was added. 24 hours post infection, cells were harvested and RNA was isolated. RNA samples were used for EBOV L-specific RT-qPCR and the copies/well were calculated using an EBOV-specific standard curve (n=1). (D) Shown are treatments with different concentrations of EBOV L-specific ASO and different conditions like pretreatment with ASO, transfection with ASO or transfection with plasmids. Huh7 cells were pre-incubated with dilutions of EBOV L-specific ASO or negative control Neg1. The next day, cells were transfected with ASOs or empty mCherry plasmid as a control. Five hours post transfection, medium was changed to ASO-containing medium. 24 hours post transfection, cells were infected with EBOV rgZ-GFP with an MOI of 0.01 for 1 hour. After infection, cells were washed to remove unbound virus and ASO-containing medium was added. 24 hours post infection cells were harvested and RNA was isolated. RNA samples were used for EBOV NP-specific RT-qPCR and the copies/well were calculated using an EBOV specific standard curve (n=1).

Fig. S2. PTO-backbone mediated inhibition of non-NPC1-specific ASOs Neg1, Neg1b and S5 compared to NPC1-specific ASO 28H. HeLa cells pretreated with the respective ASO were infected with EBOV at an MOI of 0.01. At 1 d p.i., *NPC1* (A) and EBOV (B) levels were quantified by RT-qPCR and normalized to the internal control α-tubulin. Shown is the fold change compared to untreated control (set as 100), which was calculated using the 2-ΔΔCt method. Error bars show standard deviations (n=3, each in duplicates). Duplicates were labeled with identical symbol shapes. ANOVA test was used to test for significant differences and P values were determined using Dunnett's test in GraphPad Prism 7.04 Software.

Supplemental Tables

Table S1

Antisense oligonucleotides targeting host factor Niemann-Pick C1 as well as non-specific control oligonucleotides Neg1, Neg1b and S5. Depicted are Name of ASO, mRNA binding sequence, position on mRNA, ASO length as well as ASO sequence and modification: LNA (+) and/or phosphorothioate (*). Human-specific ASOs (H) as well as cross-reactive ASOs targeting both, human and murine *NPC1* (HM), were selected.

Name	mRNA binding sequence	Position	Length	Sequence
01H	AACCAGCCGAACGCCGC	229	17	+G*+C*+G*G*C*G*T*T*C*G*G*C*T*G*+G*+T*+T
02H	ACCAGCCGAACGCCGCC	230	17	+G*+G*+C*G*G*C*G*T*T*C*G*G*C*T*+G*+G*+T
03H	GCATGACCGCTCGCGGC	273	17	+G*C*C*G*C*G*A*G*C*G*G*T*C*A*T*+G*+C
04HM	TGTTTGGTATGGAGAGT	349	17	+A*+C*+T*C*T*C*C*A*T*A*C*C*A*A*+A*+C*+A
05HM	GGAGAGTGTGGAATTGC	359	17	+G*+C*+A*A*T*T*C*C*A*C*A*C*T*C*+T*+C*+C
06HM	CTTAGTGCAGGAACT	445	15	+A*+G*+T*T*C*C*T*G*C*A*C*T*+A*+A*+G
07HM	ATGTACAATGCCTGCCG	740	17	+C*+G*+G*C*A*G*G*C*A*T*T*G*T*+A*+C*+A*+T
08HM	GCCACCAACTGGATTGA	830	17	+T*+C*A*+A*T*C*C*A*G*T*T*G*G*T*G*G*+C
09HM	TGTATGTCATCATGTGG	1077	17	+C*+C*+A*C*A*T*G*A*T*G*A*C*A*T*+A*+C*+A
10HM	GTATGTCATCATGTGG	1078	16	+C*+C*+A*C*A*T*G*A*T*G*A*C*A*+T*+A*+C
11H	GTATGCCGATTACCAC	1789	16	+G*+T*+G*G*T*A*A*T*C*G*G*C*A*+T*+A*+C
12H	CGATTACCACACGCAC	1795	16	+G*+T*+G*C*G*T*G*T*G*G*T*A*A*+T*+C*+G
13HM	CGTGGCTTGTGTTGG	1902	15	+C*+C*A*A*C*A*C*A*A*G*C*+C*+A*+C*+G
14HM	TGGACAACATCTTCA	2370	15	+T*+G*+A*+A*G*A*T*G*T*T*G*T*+C*+C*+A
15HM	TTCGCTTCTTCAAAA	2727	15	+T*+T*+T*T*G*A*A*G*A*A*G*C*+G*+A*+A
16HM	CAGAACATGGTGTGCGG	2987	17	+C*+C*+G*C*A*C*A*C*C*A*T*G*T*T*C*+T*+G
17HM	ACATGGTGTGCGGCG	2991	15	+C*+G*+C*C*G*C*A*C*A*C*C*A*+T*+G*+T
18HM	GTGTGCGGCGGCATG	2996	15	+C*+A*+T*G*C*C*G*C*C*G*C*A*+C*+A*+C
19H	AGATATTTAACGCGGC	3039	16	+G*+C*+C*G*C*G*T*T*A*A*A*T*A*+T*+C*+T
20H	GATATTTAACGCGGC	3040	15	+G*+C*+C*G*C*G*T*T*A*A*A*T*+A*+T*+C
21HM	CCTCGTCCTGGATCGA	3090	16	+T*+C*G*A*T*C*C*A*G*G*A*C*G*A*+G*+G
22HM	TTCATGACCTACCACAC	3386	17	+G*+T*+G*T*G*G*T*A*G*G*T*C*A*T*+G*+A*+A
23HM	TCATGACCTACCACAC	3387	16	+G*+T*+G*T*G*G*T*A*G*G*T*C*A*+T*+G*+A
24H	GGCGCGATATTTCTGG	3593	16	+C*+C*+A*G*A*A*A*T*A*T*C*G*C*+G*+C*+C
25H	ACATAACCAGAGCGTT	3783	16	+A*+A*+C*G*C*T*C*T*G*G*T*T*A*+T*+G*+T
26HM	GTGGAATCACACTTAC	3873	16	+G*+T*+A*A*G*T*G*T*G*A*T*T*C*C*+A*+C
27HM	ACTCAGTTACATAGG	4015	15	+C*C*T*A*T*G*T*A*A*C*T*G*+A*+G*+T
28H	AGCGCGAACGGCTTCTA	4086	17	+T*+A*+G*A*A*G*C*C*G*T*T*C*G*C*+G*+C*+T
29H	TCGGTCGGTTTACCACT	4151	17	+A*+G*+T*G*G*T*A*A*A*C*C*G*A*C*+C*+G*+A

30H	CGGTCGGTTTACCACT	4152	16	+A*+G*+T*G*G*T*A*A*A*C*C*G*A*+C*+C*+G
31H	TTGAACGTAGCGCCTG	4240	16	+C*+A*+G*G*C*G*C*T*A*C*G*T*T*+C*+A*+A
32HM	TCAGAATGTTGTAGGCC	4609	17	+G*+G*C*C*T*A*C*A*A*C*A*T*T*C*+T*+G*+A
33HM	ATGTTGTAGGCCTCATT	4614	17	+A*+A*T*G*A*G*G*C*C*T*A*C*A*+A*+C*+A*+T
34HM	TGTAGGCCTCATTAGA	4618	16	+T*+C*+T*A*A*T*G*A*G*G*C*C*T*+A*+C*+A
35HM	TGTAGGCCTCATTAGAG	4618	17	+C*+T*+C*T*A*A*T*G*A*G*G*C*C*T*+A*+C*+A
36HM	GTAGGCCTCATTAGAGC	4619	17	+G*+C*+T*C*T*A*A*T*G*A*G*G*C*C*+T*+A*+C
Neg1	N/A	N/A	18	+C*+G*+T*T*T*A*G*G*C*T*A*T*G*T*A*+C*+T*+T
Neg1b	N/A	N/A	17	+G*+T*T*T*A*G*G*C*T*A*T*G*T*A*+C*+T*+T
S5	N/A	N/A	17	+T*+T*+A*T*G*T*C*C*G*G*T*T*A*T*+T*+T*+C

Supplemental Methods

EBOV minigenome assay and EBOV trVLP assay (producer cell setting)

Huh7 (human hepatoma) cells were seeded in 12-well or 96-well format in Dulbecco's modified Eagle's medium (DMEM) (Thermo Fisher, USA, no. 21969-035) with penicillin and streptomycin 50 U/ml (Thermo Fisher, USA, no. 15070063), 2 mM glutamine (Gibco®, Thermo Fisher, USA, no. 25030024) and 10 % fetal bovine serum (Thermo Fisher, USA, no. 10270-106) at 37 °C and 5 % CO_2 for 70 % confluency next day. Three hours post seeding, cells were treated with EBOV L-specific ASO or negative control Neg1. The EBOV L-specific ASO consists of the following sequence: +A*+T*+A*A*G*G*C*A*A*T*T*T*+T*+C*+C (+, LNA; *, phosphorothioate). The next day, EBOV minigenome assay or trVLP assay (producer cell setting) was performed as described earlier [1,2]. Huh7 cells were transfected using TransIT-LT1 (Mirus Bio LLC, USA, no. MIR2306) with a plasmid containing an EBOV-specific minigenome encoding a renilla luciferase under control of a T7 promoter, a T7 polymerase as well as plasmids encoding viral proteins EBOV NP, VP35, VP30 and L (minigenome assay) or EBOV NP, VP35, VP30, L, VP24, VP40 and GP (trVLP assay, producer cell setting) A firefly luciferase reporter (Promega, USA, no. pGL4.13) was used for normalization, if technically possible. 3-4 hours post transfection, medium was changed to ASO-containing medium. 24 hours post transfection cells were lysed and used for luciferase assay (pjk, Germany). The positive control was set to 100 %.

EBOV infection assay

All work with infectious EBOV was performed in compliance with national regulations at the BSL4 laboratory of the Institute of Virology, Philipps-University Marburg.

Huh7 cells were pretreated with EBOV-specific ASO or negative control Neg1 at 2-5 hours after seeding. Oligonucleotides were either simply added to the cell culture medium or cells were transfected with ASOs. The next day, cells were infected with EBOV Makona or rgEBOV-eGFP [3,4] at an MOI of 0.01 for 1 hours in the absence of ASOs. Then, cells were washed to remove unbound virus and incubated in presence of ASO-containing medium. At 24 hours post infection, cells were harvested, and RNA was isolated using the RNeasy Mini Kit (Qiagen, Hilden, Germany; no. 74106). Viral genome copies/well were determined using EBOV L- or NP-specific primers and probes for RT-qPCR analysis [5] as well as an EBOV-specific standard curve.

Supplemental References

[1] Mühlberger E, Weik M, Volchkov VE, Klenk HD, Becker S. Comparison of the transcription and replication strategies of marburg virus and Ebola virus by using artificial replication systems. J Virol 1999;73:2333–42.

[2] Hoenen T, Groseth A, Kolesnikova L, Theriault S, Ebihara H, Hartlieb B, et al. Infection of Naive Target Cells with Virus-Like Particles: Implications for the Function of Ebola Virus VP24. Journal of Virology 2006;80:7260–4. doi:10.1128/JVI.00051-06.

[3] Hoenen T, Groseth A, Callison J, Takada A, Feldmann H. A novel Ebola virus expressing luciferase allows for rapid and quantitative testing of antivirals. Antiviral Research 2013;99:207–13. doi:10.1016/j.antiviral.2013.05.017.

[4] Ebihara H, Theriault S, Neumann G, Alimonti JB, Geisbert JB, Hensley LE, et al. In Vitro and In Vivo Characterization of Recombinant Ebola Viruses Expressing Enhanced Green Fluorescent Protein. The Journal of Infectious Diseases 2007;196:S313–22. doi:10.1086/520590.

[5] Gibb TR, Norwood DA, Woollen N, Henchal EA. Development and Evaluation of a Fluorogenic 5' Nuclease Assay To Detect and Differentiate between Ebola Virus Subtypes Zaire and Sudan. Journal of Clinical Microbiology 2001;39:4125–30. doi:10.1128/JCM.39.11.4125-4130.2001.
